# Does GPT4 dream of counting electric nodules?

**DOI:** 10.1007/s00330-023-09671-4

**Published:** 2023-04-26

**Authors:** Christian Blüthgen

**Affiliations:** 1 Institute for Diagnostic and Interventional Radiology, University Hospital Zurich, University of Zurich, Rämistrasse 100, CH-8091 Zurich, Switzerland; 2 Center for Artificial Intelligence in Medicine and Imaging (AIMI), Stanford University, Stanford, CA USA

The year 2022 was nothing short of exceptional for artificial intelligence (AI) research, particularly in the realm of generative AI. Generative AI systems learn to model training data (e.g., text, images) and can synthesize new data, without merely copying the initial data.

In November 2022, OpenAI unveiled ChatGPT [1], a large language model (LLM) designed as a chatbot, capable of processing complex text queries and producing coherent natural language outputs resembling human conversation partners. Adding to the excitement was the fact that model access was provided to a wide audience through a simple web interface. This shift towards accessible AI, combined with the model’s impressive capabilities, piqued widespread interest from clinicians, scientists, and the general public. Radiologists and other medical experts, even those with little or no technical background, can now evaluate these models for medical purposes. At the time of this writing, there are nearly 200 PubMed entries (query: “ChatGPT”) and around 7700 Google Scholar results, more than enough to warrant a closer look at the applications and limitations for our increasingly digitized field of radiology.

ChatGPT works by sequentially predicting the next parts (tokens) of its output based on the provided input and its own previous output. This autoregressive architecture is well-suited for generating text that follows a left-to-right linear structure, such as composing or summarizing a radiology report [2, 3], but can struggle with tasks that require elaborate planning ahead or discontinuous tasks, e.g., when leaps in knowledge are needed [4]. It may also be the reason why ChatGPT tends to be bad at telling new jokes.

Although ChatGPT’s underlying model (generative pretrained transformer, GPT 3.5) was pretrained on a predominantly non-medical dataset (from sources like Wikipedia, scientific articles, and news websites), and not specifically on radiology reports, it appears to have incorporated enough radiological terminology on top of general language structure to produce outputs that sound fluent and plausible. Unfortunately, the model’s outputs are not necessarily true; in fact, the model can very confidently “hallucinate” plausible sounding but blatantly wrong statements. Even more concerning, ChatGPT can double down and back up its erroneous claims with fabricated references including author lists, paper titles, hyperlinks, and even document object identifiers (DOIs). It is obvious that ChatGPT and similar LLMs are currently unfit to reliably provide medical information without human supervision.

A substantial part of ChatGPT’s success lies in its alignment process, which guides outputs with the help of a reward model that was trained on human feedback to rank outputs from a precursor model [1]. This effort led to more nuanced results and seems to have empowered rather than restricted the model. As Sam Altman, CEO of OpenAI, noted, “[Capability and alignment are] very close. Better alignment techniques lead to better capabilities, and vice versa” [5]. For radiology AI, a similar alignment process may be needed for reliably using an LLM in clinical practice. Nonetheless, extensive efforts are needed to have a medical LLM approved in most current jurisdictions [6].

Academic radiologists can already benefit from LLMs, which can assist in handling unstructured data prevalent in the medical field, summarizing research papers, and enhancing communication. All that is required are the right instructions: For example, LLMs can transfer lung nodule measurements mentioned in a radiology report into a CSV file to speed up data collection: “Collect all lung nodule measurements and return a list of the form < Side > , < Lobe > , < Segment > , < Size > with one line per nodule”. As processing large patient datasets raises privacy concerns, however, properly instructed LLMs can also aid in de-identification tasks [7]. Language barriers potentially affecting effective communication can be mitigated, as ChatGPT is multilingual and can aid non-native English speakers in translating, but also formulating their ideas with proper and concise English, fostering better understanding among researchers from different backgrounds. Apart from redacting, LLMs could also help with formatting tasks, e.g. to adapt a text to a target journal’s preferred style before submission. Lastly, LLMs can provide coding support for academic radiologists looking into programming, e.g., for generating code snippets for scientific plotting, providing debugging support, and much more.

At the current rapid pace of AI development, ChatGPT (at least its version 3.5) may soon become outdated. While LLMs are known to struggle with basic tasks like arithmetic, they can effectively overcome such shortcomings when given access to simpler, task-specific models and tools through APIs [8]. According to Bubeck et al’s 155-page report “Sparks of Artificial General Intelligence”, GPT4 can effectively use calculators and search engines, boosting its capabilities [4]. Plugins allow access to more complex AI models, like Meta’s recently introduced domain-agnostic “Segment Anything Model” (SAM) [9]. When LLMs like GPT4 capable of using tools become broadly available, the acronym GPT may receive a second meaning as **g**eneral **p**urpose **t**echnology, with the potential for far-reaching effects on the labor market [10].

For radiologists, who deal with medical images and text on a daily basis, another exciting update accompanying GPT4 are multimodal inputs (e.g., images and text), already allowing the model to describe image contents and explain them in the context of other multimodal inputs. While GPT4’s multimodal capabilities are currently restricted to a small group of researchers, other powerful vision-language models (VLM) are already available for radiology today: Fine-tuned text-to-image models are able to synthesize chest x-rays, whose appearance can be controlled through text prompts [11, 12]. Another recently released model is BiomedCLIP, a new VLM that uses large-scale multi-modal contrastive pretraining specifically for biomedical tasks [13]. The vision-language entanglement of these models can facilitate a whole range of applications, such as the creation of more capable biomedical AI systems; the generation of tailored training data to improve subgroup performance (e.g., to train less biased AI models); and providing educational examples of pathologies without having to query a hospital’s PACS.

In the title of his eponymous 1968 novel, Philip K. Dick asks whether androids, human-like artificial entities, dreamt of electric sheep, raising a whole array of philosophical questions [14]. It is an easily committed fallacy to anthropomorphize LLMs like ChatGPT and assume an “understanding”, but it is nonetheless remarkable that discussions in these directions are currently unfolding, even among seasoned AI researchers. LLMs are beginning to display (sometimes unexpected) emerging abilities and hold tremendous potential for radiology. Whether or not this will entail dreaming about counting electric nodules, for us radiologists, it does not matter as much as the fact that it will most likely, in the not-too-distant future, be possible to instruct an AI system to perform this task. We are living in the future, and the future of AI in radiology is multimodal.

## Declarations

### Guarantor

The scientific guarantor of this publication is Christian Blüthgen.

### Statistics and biometry

No complex statistical methods were necessary for this paper.

### Methodology

• commentary
